# Influence of the Timing of Gender Dysphoria Presentation and Suicidal Behaviors on Internalizing Symptoms, Emotion Dysregulation, and Body Investment in Adolescents Seeking Gender Affirmation

**DOI:** 10.3390/pediatric17020037

**Published:** 2025-03-17

**Authors:** Maria Giuseppina Petruzzelli, Federica Annecchini, Flora Furente, Fabio Tarantino, Valeria Carruolo, Elisabetta Lavorato, Maria Pia Riccio, Andrea De Giacomo, Emilia Matera

**Affiliations:** 1 Translational Biomedicine and Neuroscience Department (DiBraiN), University of Bari “Aldo Moro”, 70124 Bari, Italy; maria.petruzzelli@uniba.it (M.G.P.); flora.furente@uniba.it (F.F.); tarantinofabio295@gmail.com (F.T.); valeriacarruolo@gmail.com (V.C.); andrea.degiacomo@uniba.it (A.D.G.); 2 Department of Medical and Translational Sciences, Child Neuropsychiatry, Federico II University, Corso Umberto I 40, 80138 Napoli, Italy; piariccio@gmail.com; 3 Psychiatry Unit, Azienda Ospedaliero-Universitaria Policlinico di Bari, University of Bari “Aldo Moro”, 70100 Bari, Italy; lav.elisabetta.72@gmail.com; 4 Department of Regenerative and Precision Medicine and Jonic Area (DiMePRe-J), University of Bari “Aldo Moro”, 70124 Bari, Italy

**Keywords:** gender dysphoria, adolescents, emotional dysregulation, body image, self-harm behaviors, internalizing disorders

## Abstract

Studies have consistently shown that gender-diverse youth experience higher rates of internalizing disorders and self-harm behaviors (SH) compared to their cisgender peers. However, there is limited research on how body investment and emotion regulation influence these symptoms, especially in relation to the age of gender dysphoria (GD) presentation. Objectives: This study aimed to explore the relationship between the timing of GD presentation (early vs. late) and psychological distress in adolescents seeking gender affirmation (GA), specifically focusing on internalizing symptoms, emotion regulation, and body investment. The study also investigated how SH during the year preceding the request for gender affirmation might have impacted these factors. Methods: On a total of 80 adolescents (mean age: 14.88 years) at their first request for GA, participants were divided into two groups: early-presentation GD (EP-GD; mean age: 14.93 years) and late-presentation GD (LP-GD; mean age: 14.83 years). Among the sample, 60% exhibited SH. Internalizing symptoms, emotion regulation, and body investment were assessed using the Youth Self-Report (YSR), the Difficulties in Emotion Regulation Scale (DERS), and the Body Investment Scale (BIS). Results: Results revealed that LP-GD adolescents had significantly higher emotion dysregulation (ED), particularly in the Strategies domain of the DERS (*p* = 0.040), and more social problems in the YSR (*p* = 0.047) compared to EP-GD ones. SH were associated with higher internalizing symptoms, including anxiety, withdrawal, and somatic complaints (*p* < 0.03), as well as increased body dissatisfaction, particularly in the BIS Care and Protection dimensions (*p* = 0.044; *p* = 0.034). Conclusions: These findings supported the hypothesis that LP-GD adolescents and youths with a history of SH showed more pronounced emotion regulation difficulties and internalizing symptoms, further emphasizing the need for early intervention programs targeting both GD and co-occurring mental health problems.

## 1. Introduction

Gender dysphoria (GD) is defined by the Diagnostic and Statistical Manual of Mental Disorders (DSM-5-TR) as the marked incongruence between an individual’s experienced or expressed gender and the gender assigned at birth, causing significant distress or impairment in important areas of functioning [1]. Clinically significant distress is a key element of the diagnosis of GD [2] and often manifests in various forms, including emotional, social, and psychological challenges. Gender-related discomfort can manifest itself differently depending on age and developmental stage. Specifically, the DSM-5-TR describes GD in children and adolescents distinctly, highlighting how symptoms and expressions of distress can vary according to gender-related experiences at each age of presentation [1]. In summary, the emphasis shifts from discomfort related to gender roles and behaviors in children to discomfort related to physical changes and body perception in adolescents who may struggle with the complexities of both their gender identity and the physical, emotional, and psychological changes typical of this age, potentially exacerbating the condition of distress [3]. Awareness of gender incongruence is accompanied by a negative emotional investment in the body as well as a broader difficulty in regulating and managing negative emotions; both of these dimensions may play a significant role in the onset of internalizing symptoms [4,5]. Body investment is a multidimensional construct that refers to the cognitive, behavioral, and emotional importance of the body in one’s self-evaluation so that body image, body care, body protection, and body touch [6] would play a role in the prediction of self-harm behaviors (SH) including non-suicidal self-harming injuries (NSSI), suicide (SB) and suicide attempts (SA) [7]. Furthermore, the current literature highlights the well-established association between SH and greater degrees of emotion dysregulation (ED), a transdiagnostic factor that influences a variety of mental health conditions, both for children and adolescents. ED involves an inability to manage intense emotions, leading to quick emotional reactions like anger, anxiety, or depression [8,9,10]. In adolescents, ED can significantly contribute to psychological distress [11] including an increased risk of SH, as these individuals may resort to maladaptive coping strategies to manage overwhelming negative emotions [12]. For youth with GD, gender-related distress can amplify ED, making it harder for these adolescents to regulate emotions [13]. Recent literature data support the idea that adolescents who experience gender incongruence later in life probably have fewer opportunities to adapt to such emotional and social burdens, while adolescents with early onset of GD, who had likely developed coping mechanisms precociously, were generally better equipped to manage their distress when they sought gender-affirming treatment [14,15]. The literature on early- and late-onset gender dysphoria (GD) is still limited, with only a few studies addressing the differences between these presentations in terms of clinical outcomes and mental health challenges. One concept that has gained attention in recent years is “rapid onset gender dysphoria” (ROGD), which refers to adolescents who present with GD symptoms seemingly without a prior history of gender diversity in childhood [16]. This clinical presentation has gained international recognition, contributing to the understanding of this phenomenon [17,18,19]. The World Professional Association for Transgender Health (WPATH) has also acknowledged the increasing number of adolescents seeking care who have not previously expressed gender diversity. However, there is still a significant gap in the literature regarding the clinical differences between early- and late-onset GD, particularly in terms of mental health outcomes, such as depression, anxiety, and suicidality. Several studies have consistently shown that internalizing disorders, including depression and anxiety, as well as psychological distress and SH are significantly more prevalent in gender-diverse youth compared to their cisgender peers [19,20,21,22], but to our knowledge, little data exist about body investment and emotion regulation in adolescents with GD, despite the role that these dimensions may have in determining SH. Furthermore, there is still limited research on how emotion regulation and body investment influence symptom expression, particularly with respect to the age of presentation of GD. The hypothesis of this study was that the history of GD presentation—whether it manifests early in childhood or during adolescence—may be a significant factor in modulating psychological distress observed at the time of the request for gender affirmation (GA). Also, the presence or absence of SH may serve as an important marker of distress, with the hypothesis that exhibiting SH prior to initiating the GA process could correlate with emotional regulation problems, higher levels of internalizing symptoms, and more pronounced body investment difficulties. Specifically, the study will examine the key dimensions of emotional regulation, internalizing symptoms, and body investment, using established standardized tools such as the Youth Self-Report (YSR), the Difficulties in Emotion Regulation Scale (DERS), and the Body Investment Scale (BIS). The aim was to clinically characterize a sample of 80 adolescents at the time of their first request for GA to investigate whether differences exist in those dimensions based on the presentation of GD (early vs. late) and the presence of self-harm (SH). Differentiating between these presentations is crucial in tailoring interventions and addressing the underlying psychological distress in adolescents. This distinction could also provide insights into how gender dysphoria evolves over time and how best to support individuals at different stages of their gender identity development [23].

## 2. Materials and Methods

The study involved a retrospective review of medical records of adolescent patients, up to the age of 18, at their first request for GA and referred to the Child and Adolescent Neuropsychiatry Unit at the Translational Biomedicine and Neurosciences (DiBraiN) Department, University of Bari, Italy, over a four-year period (October 2018 to October 2024). Participants were included if they met the diagnostic criteria for GD according to the DSM-5-TR [1], based on medical history, clinical observation, and specialized evaluation by physicians and psychologists of the Child and Adolescent Neuropsychiatry Unit and the GD Psychiatry Service of the same hospital. Exclusion criteria included a formal diagnosis of intellectual development disorder and failure to complete the required study protocols. All participants underwent a clinical global assessment, which examined the following: the timing of presentation and signs suggestive of GD, the history of psychological/psychiatric symptoms, and the presence of the following SH: (a) non-suicidal self-injury (NSSI), defined as self-inflicted, intentional injury to the body with the expectation that the harm would be minimal; (b) suicidal ideation, defined as recurring thoughts or reflections about the possibility of ending one’s life; and (c) suicide attempt, defined as a deliberate act with the conscious intention to end one’s life (ICD-11), either alone or in combination with other behaviors. Additionally, the assessment included the evaluation of psychiatric comorbidities based on DSM-5-TR criteria [1]. The data extraction from the clinical records was performed by trained research assistants who followed a standardized protocol to ensure consistency across cases. To ensure accuracy, all data were cross-checked by a second reviewer, and discrepancies were resolved by consulting the primary clinicians involved in the patient’s care. Based on the anamnestic data and clinical features of GD, patients were categorized into the EP-GD (early presentation) group if their GD symptoms began in childhood, and the LP-GD (late presentation) group if GD symptoms emerged in adolescence. Patients were further classified into the SB-GD group if SH were present in their medical history during the year preceding the request for GA, or into the NSB-GD group if no such behaviors were reported. Self-harm behaviors in this study were collected through detailed anamnesis, with participants providing information about their personal history of SH. These behaviors were classified according to the definition provided in the ICD-11. Standardized criteria were applied consistently across all participants to ensure uniformity in classification. The assessment of ED was conducted using the Italian version of the DERS, a 36-item self-report questionnaire, assessing six relevant domains of ER abilities [24,25]. Emotional investment in the body was assessed using the Italian translation of the BIS, a 24-item self-report measure [26], which has been used in scientific literature [6,27]. The BIS four-factor solution has demonstrated a good ability to differentiate between suicidal and non-suicidal adolescents [28]. Additionally, the study of internalizing symptoms was carried out using the YSR [29] in its Italian version [30]. This study focused on the following behavioral scales: Anxiety/Depression, Withdrawal/Depression, Somatic Complaints, and Social Problems, as well as the total internalizing scale. More details on the structure, clinical, and statistical validity of these questionnaires can be found in previous publications by this research group within the same research project (ED-AG, study number 6888, prot. 0063972 18 July 2022) [4,31].

All variables were recorded in structured forms specifically designed for this research. Data analyses were conducted using JASP software 0.19.2 version [32]. Sociodemographic and clinical characteristics of both groups were presented as frequencies and counts, while quantitative data, such as age and psychometric questionnaire scores, were reported as means, standard deviations (SD), medians, and interquartile ranges (IQR). Qualitative data were reported using descriptive statistics, while differences in psychometric scores between groups were compared using the independent *t*-test for unpaired groups or the Mann–Whitney test, depending on the distribution of the data, which were assessed using the Shapiro–Wilk test. The level of significance was set at *p* < 0.05. To provide a more comprehensive understanding of the results, effect sizes (Cohen’s d) were reported alongside *p*-values, allowing for a clearer interpretation of the magnitude and clinical significance of the observed differences.

## 3. Results

### 3.1. Demographic and Clinical Features

A total of 80 GD adolescents, enrolled consecutively between 2018 and 2024, were divided in the groups of EP-GD (mean age: 14.93 years) and LP-GD (mean age: 14.83 years). In the EP-GD group, 11 adolescents were assigned male at birth (AMAB), and 34 were assigned female at birth (AFAB). In the LP-GD group, 5 adolescents were AMAB, and 30 (85.7%) were AFAB. In the whole sample, 27.5% of adolescents exhibited multiple psychiatric comorbidities, in particular depression and anxiety, and 60% had SH in the previous year. The sociodemographic and clinical features of the sample are shown in Table 1.

#### 3.1.1. Comparison Between Early-Presentation and Late-Presentation Groups

Results obtained by comparing the internalizing dimension through YSR, emotion regulation through DERS, and self-body investment through BIS between groups classified by GD presentation onset are shown in Table 2.

Between EP-GD and LP-GD groups, statistically significant differences in the DERS total score (*p* = 0.017) and in the Strategies subdomain (*p* = 0.040) and in social problems (*p* = 0.047) measured by the YSR were found.

#### 3.1.2. Comparison Between Self-Harm and Non-Self-Harm Groups

Results obtained by comparing the internalizing dimension through YSR, emotion regulation through DERS, and self-body investment through BIS between groups classified by the presence of SH are shown in Table 3.

Statistically significant differences were found in YSR internalizing symptoms, with higher scores in the SB group in all investigated dimensions except for social problems. Additionally, significant differences were observed in the DERS total score (*p* = 0.021), as well as in the Strategies (*p* = 0.006) and Non-Acceptance (*p* = 0.003) subdomains. Finally, the SB group showed significantly higher scores in the Care and Protection dimensions of BIS (*p* = 0.044; *p* = 0.034).

## 4. Discussion

There is growing evidence of an increase in the frequency of presentation to gender affirmation services, alongside a notable shift in the natal sex of referred cases, with individuals assigned female at birth now comprising the majority. However, limited data are available on the age of onset of gender dysphoria (GD) in these populations. The available data from a systematic review of adolescent gender dysphoria literature suggest that the average age at referral is around 13 years, with the assessment typically occurring at 15 years. This lack of detailed information on the age of onset remains a significant gap in the current literature [33,34,35]. Several recent studies investigated how the trajectory of GD (i.e., whether it began in childhood or adolescence) influenced psychological outcomes [36] such as depression, anxiety, and overall distress when individuals sought gender-affirming care. Some of these highlighted that children who exhibited consistent and persistent GD from a young age (before puberty) were generally better adjusted psychologically by the time they reached adolescence [37,38] and were seeking gender-affirming care. In contrast, those whose GD emerged later, during adolescence, often exhibited more severe psychological distress [3], including higher levels of depression, anxiety, and suicidal ideation at the time of seeking care [14,15,37]. However, most of these studies did not directly compare adolescents with GD seeking gender reassignment by distinguishing between GD presentation in childhood or adolescence. The dimensions of ED and body investment are aspects of psychopathological relevance in adolescence and, specifically, in adolescents who experience a condition of distress related to GD [4]. Having made this premise according to previous research, the hypothesis of this study was that the history of gender dysphoria presentation—whether it manifested early in childhood or during adolescence—was a significant factor in modulating psychological distress observed at the time of the request for gender reassignment. Furthermore, SH might have served as an important marker of distress, and, in addition to the timing of awareness of gender incongruence, also exhibiting SH prior to initiating gender affirmation processes could have correlated with emotional regulation difficulties, higher levels of internalizing symptoms, and more pronounced body investment difficulties [23,33]. The first result of this study was that LP-GD adolescents suffered from greater ED, particularly in the Strategies domain of the DERS, suggesting that adolescents who experienced GD from an early age might have developed more adaptive emotion regulation strategies over time, perhaps due to earlier exposure to the social and psychological challenges related to gender identity. In contrast, those with later presentation of GD struggled more with adopting effective coping strategies to handle the emotional challenges associated with GD, especially in the context of adolescence [4]. In addition to emotion regulation difficulties, the LP-GD group also reported higher scores in the social problems domain of the YSR supporting the fact that adolescents with late-presentation GD might have faced more significant challenges in social interactions [36], likely due to the later realization of their gender identity, which could result in social alienation and difficulty relating to peers. Interestingly, the absence of significant differences in body investment between the EP-GD and LP-GD groups aligned with previous studies, which suggested that the body dissatisfaction and the psychological impact of pubertal changes affected both early- and late-onset GD adolescents. However, the lack of statistical significance in body investment might have also been due to the measurement tools used, as the BIS measures only specific aspects of body image and might not have fully captured the complex ways in which GD adolescents experienced body dysphoria. Another result of this study was the high prevalence of SH in the year preceding the request for gender affirmation, underscoring the urgency of addressing mental health issues in this population. The presence of SH was significantly associated with more severe internalizing symptoms across all domains measured by the YSR, including anxiety, withdrawal, and somatic complaints. Individuals with GD who exhibited SH prior to seeking gender-affirming care might have been grappling with a lack of emotional regulation skills. In our study, the SB-GD group exhibited significantly higher levels of ED, particularly in the total DERS score and in the Non-Acceptance and Strategies domains. These results suggested that adolescents engaging in SH might have had greater difficulty accepting and regulating their emotions, leading them to engage in maladaptive coping strategies [39]. Additionally, SH were associated with significantly higher scores in the BIS, specifically in the Care and Protection domains suggesting that adolescents who engage in self-harm might have had a more conflicted relationship with their bodies. The elevated scores in these BIS dimensions could have reflected a desire to “control” the body in response to the distressing experience of GD. [4]. Such findings have important clinical implications. Globally considered, these results suggest that adolescents with gender incongruence may be faced with SH when they have a bad protective attitude towards the body, rather than when they have difficulties in other domains of the multidimensional construct of BIS. This is of considerable clinical importance and requires certain therapeutic attention, especially during the adolescent years [40], because the coexistence of emotion dysregulation and bad body protective attitude [41], may increase the risk of self-injurious and suicidal behavior [42]. However, the design of our study does not allow us to definitively address the question of whether the presence of suicidal behavior is accompanied by a greater overall burden of comorbidities or if it is more closely associated with the psychopathology related to GD itself. Further studies with longitudinal designs would be necessary to explore these relationships in more detail. The differences observed between early- and late-onset gender dysphoria (GD) in this study could be further understood by considering broader contextual factors, including family support, social acceptance, and prior mental health care. In the case of early-onset GD, the path to self-awareness often occurs gradually, allowing more time for family members to adjust, offer support, and accept the individual’s gender identity. Similarly, social acceptance tends to evolve more progressively. This extended period of adjustment likely contributes to the greater psychological resilience seen in individuals with early-onset GD at the time of seeking gender affirmation. Literature supporting this perspective suggests that individuals with a history of early presentation of GD benefit from more consistent family and social support, which in turn positively impacts mental health outcomes when they pursue gender-affirming treatment [36]. In contrast, adolescents with late-onset GD often experience more acute distress, as they may face challenges related to delayed recognition of their gender identity, which could result in limited or delayed social and familial support. This is reflected in the results of this study, where a significant difference was observed in social issues, confirming that adolescents with late-onset GD tend to experience more challenges in this area. Additionally, cultural factors—such as societal norms, attitudes towards gender diversity, and local healthcare access—play a significant role in shaping the experiences of gender-diverse individuals, influencing both their mental health outcomes and the type of support they receive. Adolescents with late-onset GD might require specialized support to address emotion regulation difficulties, especially those involving maladaptive coping strategies that emerged during adolescence. Given the emotional and social challenges associated with late-presenting GD, therapeutic interventions should focus on helping these adolescents process their gender identity in a supportive and validating environment. Additionally, the high prevalence of SH in adolescents with GD underscores the need for early intervention programs that target both the specific psychological aspects of GD and the associated mental health issues, such as anxiety, depression, and self-harm [43].

## 5. Conclusions

Given the high prevalence of suicidality, depression, and anxiety among transgender individuals, understanding how early and late presentation of GD impacts mental health is clinically significant. This study highlights the psychological challenges faced by adolescents with gender dysphoria (GD), specifically emphasizing the impact of GD onset timing. Adolescents with late-onset GD (LP-GD) exhibited greater difficulties in emotion regulation (ED), particularly in the Strategies domain. This finding underscores the need for early psychological interventions to address emotion regulation, particularly for LP-GD adolescents who may have had less time to develop adaptive coping strategies. This distinction could also provide insights into how gender dysphoria evolves over time and how best to support individuals at different stages of their gender identity development.

Additionally, the high prevalence of self-harm (SH) in the year preceding the request for gender-affirming care underscores the urgency of addressing mental health concerns in this population. In conclusion, these findings suggest that early intervention programs should focus on addressing emotion regulation difficulties, maladaptive coping strategies, and mental health issues like anxiety, depression, and self-harm. Future research should continue to investigate these factors and explore the role of family support, social acceptance, and socio-cultural influences in shaping the psychological experiences of adolescents with GD.

## 6. Limitations and Future Directions

This study has some limitations, including the relatively small sample size and the retrospective nature of the data collection, which limits the ability to establish causal relationships. The division into subgroups further limits the statistical power, and the results should therefore be considered preliminary. Additionally, the study lacks a comparison group. Future research should involve larger, more diverse samples, and longitudinal studies are needed to further explore the long-term psychological outcomes of adolescents with gender dysphoria. Furthermore, it would be valuable to examine the role of socio-cultural and family factors in shaping the psychological experiences and coping mechanisms of adolescents with GD. Understanding how these external factors influence the development of GD and related mental health concerns could inform more effective, tailored interventions. Future studies could also examine the relationship between self-esteem and gender dysphoria, particularly given its known links to internalizing symptoms and identity formation during adolescence.

## Figures and Tables

**Table 1:** Sociodemographic and clinical features of adolescents seeking GA (n = 80).

Clinical Features	Count (Frequencies)
Age at GA Request (mean ± SD)	14.9 ± 1.8 y
Gender Assigned at Birth	
AMAB	16 (20.0%)
AFAB	64 (80.0%)
GD Presentation	
Early Presentation	44 (55.0%)
Late Presentation	36 (45.0%)
SH in the Previous Year	
Absent	32 (40.0%)
Present	48 (60.0%)
Non-Suicidal Self-Injury	40 (50.0%)
Suicidal Ideation	37 (46.2%)
Suicide Attempt	14 (17.5%)
Psychiatric Comorbidities	
No Comorbidities	23 (28.7%)
Multiple Comorbidities	22 (27.5%)
Depressive Disorders	17 (21.2%)
Anxiety Disorders	11 (13.7%)
Obsessive-Compulsive Disorder	2 (2.5%)
Disorders related to traumatic and stressful events	1 (1.2%)

**Table 2:** Comparison between scores of YSR, DERS, and BIS between EP-GD and LP-GD groups. (YSR: Youth Self-Report; DERS: Difficulties in Emotion Regulation Scale; BIS: Body Investment Scale).

	EP-GD Mean (SD)	EP-GD Median (IQR)	LP-GD Mean (SD)	LP-GD Median (IQR)	*p*-Value	Cohen’s d
YSR-Int TOT	65.7 (11.4)	64.0 (13.0)	69.4 (11.0)	65.0 (19.0)	0.280	0.32
YSR-Anxious	66.9 (11.2)	65.5 (13.8)	71.5 (12.8)	68.0 (19.0)	0.180	0.39
YSR-Withdrawn	66.5 (12.3)	64.0 (16.0)	70.7 (15.1)	66.0 (26.5)	0.210 *	0.28
YSR-Som Complain	61.9 (10.6)	60.5 (12.5)	62.4 (9.0)	63.0 (14.0)	0.860 *	0.05
YSR-Social	61.6 (8.9)	60.0 (13.3)	66.6 (9.9)	68.0 (13.5)	0.047	0.53
DERS Total	102.3 (26.9)	103.5 (36.5)	117.8 (27.7)	116.0 (33.0)	0.017	0.59
DERS Non-Accept	14.9 (6.5)	14.0 (10.0)	17.5 (7.5)	16.0 (12.0)	0.114 *	0.35
DERS Goals	18.3 (5.2)	19.0 (5.5)	20.3 (4.1)	21.0 (5.3)	0.054	0.38
DERS Impulse	15.4 (7.1)	13.0 (10.3)	19.0 (7.6)	18.0 (13.3)	0.051	0.50
DERS Awareness	17.0 (5.4)	16.0 (8.3)	17.7 (6.2)	17.0 (12.0)	0.712	0.12
DERS Strategies	22.2 (7.5)	21.5 (11.0)	25.9 (8.2)	26.5 (11.5)	0.040	0.53
DERS Clarity	14.8 (4.8)	15.0 (7.0)	16.8 (5.1)	17.0 (8.0)	0.077	0.39
BIS-C	18.0 (4.7)	16.5 (7.5)	17.1 (7.3)	17.5 (7.8)	0.483	0.14
BIS-T	17.5 (5.6)	18.0 (8.0)	17.1 (7.3)	17.5 (12.5)	0.667	0.05
BIS-P	20.6 (4.8)	21.0 (5.0)	19.1 (5.7)	19.0 (9.5)	0.123	0.27
BIS-I	13.8 (5.2)	13.0 (5.0)	14.4 (6.1)	12.0 (6.0)	0.659	0.12

* The comparison is made by *t*-test for unpaired data.

**Table 3:** Comparison between scores of YSR, DERS, and BIS between NSB-GD and SB-GD groups. (YSR: Youth Self-Report; DERS: Difficulties in Emotion Regulation Scale; BIS: Body Investment Scale).

	NSB-GD Median (IQR)	NSB-GD Mean (SD)	SB-GD Median (IQR)	SB-GD Mean (SD)	*p*-Value	Cohen’s d
YSR-Int TOT	60.0 (7.5)	62.4 (8.2)	73.0 (17.3)	70.5 (11.9)	<0.001	0.82
YSR-Anxious	62.0 (9.5)	63.4 (9.6)	70.0 (18.5)	72.5 (12.3)	0.002	0.85
YSR-Withdrawn	61.0 (11.0)	62.3 (10.3)	69.0 (26.0)	72.3 (14.3)	0.002 *	0.83
YSR-Som Complain	58.0 (10.0)	59.0 (8.0)	64.0 (13.0)	64.1 (10.5)	0.032 *	0.56
YSR-Social	61.0 (13.0)	62.0 (9.1)	65.0 (12.0)	65.0 (9.9)	0.244	0.32
DERS Total	99.0 (49.5)	99.0 (28.7)	116.0 (41.5)	115.3 (26.1)	0.021	0.43
DERS Non-Accept	11.0 (7.0)	13.0 (6.1)	18.0 (10.5)	17.8 (7.0)	0.003	0.71
DERS Goals	18.0 (8.0)	18.3 (5.7)	20.0 (5.0)	19.7 (4.2)	0.221	0.37
DERS Impulse	14.0 (11.0)	15.2 (6.8)	18.0 (14.0)	18.0 (7.6)	0.118	0.39
DERS Awareness	15.0 (10.0)	15.7 (5.6)	18.0 (9.0)	18.3 (5.6)	0.051	0.43
DERS Strategies	20.0 (11.0)	20.4 (7.2)	27.0 (13.0)	25.8 (7.7)	0.006	0.60
DERS Clarity	14.0 (7.0)	14.7 (5.4)	16.0 (7.5)	16.2 (4.7)	0.228	0.35
BIS-C	19.5 (7.5)	18.9 (4.2)	16.0 (7.5)	16.8 (4.9)	0.044	0.43
BIS-T	19.0 (11.0)	19.0 (5.7)	21.5 (6.0)	16.2 (6.5)	0.063	0.33
BIS-P	15.0 (7.5)	16.2 (4.6)	20.0 (8.5)	21.5 (4.8)	0.034	1.04
BIS-I	13.0 (7.5)	14.5 (6.2)	12.0 (5.0)	13.7 (5.1)	0.569 *	0.11

* The comparison is made by *t*-test for unpaired data.

## Data Availability

All relevant data are within this paper.
